# Evolution of the modular, disordered stress proteins known as dehydrins

**DOI:** 10.1371/journal.pone.0211813

**Published:** 2019-02-06

**Authors:** Andrew C. Riley, Daniel A. Ashlock, Steffen P. Graether

**Affiliations:** 1 Graduate Program in Bioinformatics, University of Guelph, Guelph, Ontario, Canada; 2 Department of Molecular and Cellular Biology, University of Guelph, Guelph, Ontario, Canada; 3 Department of Mathematics & Statistics, University of Guelph, Guelph, Ontario, Canada; University of Lausanne, SWITZERLAND

## Abstract

Dehydrins, plant proteins that are upregulated during dehydration stress conditions, have modular sequences that can contain three conserved motifs (the Y-, S-, and K-segments). The presence and order of these motifs are used to classify dehydrins into one of five architectures: K_n_, SK_n_, K_n_S, Y_n_K_n_, and Y_n_SK_n_, where the subscript n describes the number of copies of that motif. In this study, an architectural and phylogenetic analysis was performed on 426 dehydrin sequences that were identified in 53 angiosperm and 3 gymnosperm genomes. It was found that angiosperms contained all five architectures, while gymnosperms only contained K_n_ and SK_n_ dehydrins. This suggests that the ancestral dehydrin in spermatophytes was either K_n_ or SK_n_, and the Y-segment containing dehydrins first arose in angiosperms. A high-level split between the Y_n_SK_n_ dehydrins from either the K_n_ or SK_n_ dehydrins could not be confidently identified, however, two lower level architectural divisions appear to have occurred after different duplication events. The first likely occurred after a whole genome duplication, resulting in the duplication of a Y_3_SK_2_ dehydrin; the duplicate subsequently lost an S- and K- segment to become a Y_3_K_1_ dehydrin. The second split occurred after a tandem duplication of a Y_1_SK_2_ dehydrin, where the duplicate lost both the Y- and S- segment and gained four K-segments, resulting in a K_6_ dehydrin. We suggest that the newly arisen Y_3_K_1_ dehydrin is possibly on its way to pseudogenization, while the newly arisen K_6_ dehydrin developed a novel function in cold protection.

## Introduction

Due to their sessile nature, plants have evolved various methods for responding to biotic and abiotic stresses. Contact with abiotic (environmental) stresses can cause severe damage to plants, which can result in crop loss, growth impairment, and even death [1,2]. Dehydration, itself a significant abiotic stress in plants, can take many forms, such as drought, cold, and high salinity. Under such conditions, plants face numerous problems, including mechanical impairment, alterations in turgor pressure, and loss of cell integrity [1]. A group of proteins, known as dehydration proteins (dehydrins), have their expression correlated with dehydration stress and protection [3]. Dehydrins are a member of a protein family known as the late embryogenesis abundant (LEA) proteins [3–7]. The exact *in vivo* biochemical function of dehydrins is currently unknown, however *in vitro* experiments have given clues as to how they can protect plants. Experiments have shown that dehydrins may have roles in enzyme cryoprotection [8–14], membrane protection [15–17], and protection against reactive oxygen species [18–20].

Dehydrin sequences are highly hydrophobic, with over 50% of the residues being charged or polar, and over 25% being alanine or glycine [21]. Not surprisingly, these proteins are classified as being intrinsically disordered proteins (IDPs), which has been experimentally demonstrated for dehydrins by several circular dichroism and NMR studies [22–26]. IDPs that are fully disordered, as is the case for dehydrins, have very little secondary structure and almost no tertiary structure. As such, they are described as “protein clouds” [27] or resembling “cooked spaghetti” [28]. In the case of dehydrins, their disordered nature is important in their cryoprotective function, likely related to their large hydrodynamic radius compared to ordered proteins with the same number of residues [25], and their ability to bind large quantities of water molecules and ions [29].

Since the biological function of dehydrins has not yet been fully established, and they do not have regular tertiary structure, dehydrins are technically defined by the presence of a motif known as the K-segment, which is a lysine-rich motif than can be defined by the sequence [XKXGXX(D/E)KIK(D/E)KXPG] [21,30]. Although the K-segment is used to define a dehydrin, no one position in the K-segment is completely conserved [31]. There are two other common motifs found in dehydrins: the Y-segment ([D(D/E)(Y/H/F)GNPX], where the X is mostly hydrophobic), and the S-segment ([LHR(S/T)GS_4-6_(S/D/E)(D/E)_3_]) [30]. The role of the Y-segment is unknown, however due to its similarity to the nucleotide binding site of the *Escherichia coli* chaperone protein GroES, it has been suggested that the Y-segment may have a similar function, although our work suggests otherwise (Boddington & Graether, unpublished results). In the case of the S-segment, it has been found that, when phosphorylated, it can transfer dehydrins to the nucleus from the cytosol [32]. In between the conserved motifs are the ϕ-segments, which are stretches of sequence with a variable length, and while not conserved, they do contain mostly small, polar and charged amino acids. The conserved segments are used to classify dehydrins into five major architectures: K_n_, SK_n_, K_n_S, Y_n_K_n_, and Y_n_SK_n_, where the subscript n indicates a segment that may occur multiple times, and the order represents how they are arranged in the protein sequence [30].

The roles and the cellular localization of the different dehydrin architectures is not fully known and understood, but some patterns in the upregulation under abiotic stress have been identified. In a review by Graether and Boddington [31], it was found that the majority of K_n_, SK_n_, and K_n_S dehydrins were upregulated during cold stress, with some also being upregulated under desiccation and salt stress. Y_n_SK_n_ dehydrins were only upregulated during desiccation and salt stress. Of the two Y_n_K_n_ they compared, one was upregulated under cold stress, and the other was upregulated by desiccation and salt stress [31]. In terms of localization, most dehydrins are found in both the nucleus and the cytoplasm, however SK_n_ dehydrins have been found near the plasma membrane [33,34], and one K_n_S dehydrin has been found in the mitochondria [16]. The localization of many S-segment containing dehydrins to the nucleus supports the proposed role of the S-segment. However both K_n_ and Y_n_K_n_ dehydrins have also been found in the nucleus, suggesting the S-segment may not be the sole sequence element required for a dehydrin to be moved to the nucleus [35–37].

Plant evolution has led to diverse numbers and architectures of dehydrins in different plant species. Of the 35 angiosperm species examined by Malik *et al*., all of them contained at least one SK_n_ dehydrin [21]. Thirty-three species contained at least one Y_n_SK_n_ dehydrin, 13 species contained at least one Y_n_K_n_ dehydrin, 15 species contained at least one K_n_ dehydrin, and 23 species contained at least one K_n_S dehydrin [21]. The number of each type of architecture varied, with some species having as many as nine dehydrins with the same architecture. Understanding how and when these architectures arose is important in gaining an improved knowledge of dehydrin function.

The evolution of dehydrins has been previously studied in *Arabidopsis thaliana* [6,38], *Malus domestica* [39], *Solanum tuberosum* [40], *Brassica napus* [41], *Populus trichocarpa* [42], *Hordeum vulgare* [43], and *Oryza* [44]. However, they focused primarily on the evolution of dehydrins in one species or genus. Studying the evolution of dehydrins as a whole can provide broader insight into their origins and role in plants. Understanding which dehydrins were retained after duplication, and how they changed, is important in understanding their function. We investigate here how different dehydrin architectures may have arisen, and been retained or lost, after various duplication events.

## Materials and methods

### Dehydrin sequence database

Dehydrin sequences were collected from the Phytozome 12 database using Biomart to filter for sequences with the dehydrin PFAM designation (PF00257) [45–47]. Duplicate sequences, alternative transcripts, sequences that did not start with methionine, sequences with ambiguous amino acids, or sequences from early released genomes were removed. A K-segment motif was generated using Multiple EM Motif Elicitation (MEME)[48] on the sequences collected by Malik *et al*. to create a K-segment search expression [21]. MEME is a tool that can be used to find ungapped motifs in a set of sequences [48]. Motif Alignment & Search Tool (MAST) was then used to search for the K-segment in the first set of sequences collected using the dehydrin PFAM description; only sequences that had the motif with a combined *p*-value of <10^−5^ were kept [49]. The retained sequences were used to search for more sequence in the Phytozome using BLASTP; matches with an *E*-value <1 were kept [45]. The same search for the K-segment using MEME was repeated, and the newly refined collection of sequences was used to search for more sequences in the Phytozome using BLASTP again. This process was repeated until no new sequences were collected [45]. The same search was performed in the ConGenIE database [50] to search for dehydrins in *Picea abies* and *Pinus taeda*, and in the Giga database to search for dehydrins in *Ginkgo bilboa* [51]. Three sequences in the Phytozome and two sequences in the ConGenIE database were detected that had the PFAM description for reticulon-like proteins; these sequences were not added to the database.

### Dehydrin architecture annotation

The sequences in the dehydrin database were run through the MEME software, to search for 10 different motifs; the rest of the parameters were left at the default settings [48]. The detected motifs were used to mark sequences as belonging to the K_n_, SK_n_, Y_n_K_n_, Y_n_SK_n_, or K_n_S architectures [21]. The S-segment in some K_n_S dehydrin were not detected by MEME; after visual inspection of all of the sequences, those that matched the regular expression [S(SGD)SDSD] were annotated as K_n_S dehydrins.

### Multiple sequence alignments and phylogenetic tree construction

The dehydrin protein sequences were aligned using MUSCLE on Emboss using the default parameters [52,53]. Multiple sequence alignments (MSA) were visualized using Seaview [54]. The phylogenetic tree was constructed using an MSA that included only dehydrins from spermatophytes in order to prevent very divergent dehydrins from reducing the quality of the alignment. PAL2NAL was used to convert the protein alignment into a codon alignment [55]. Next, the codon alignment was run through Model Generator to obtain parameters for use in a maximum-likelihood tree [56]. The maximum-likelihood phylogenetic tree was constructed using RAxML, with the general time reversible model for the substitution model and the GAMMA model with invariant sites for rate heterogeneity [57]. One hundred rapid bootstrap samplings were run. The resulting tree was visualized using MEGA X [58].

### Dehydrin ortholog detection

To check if a dehydrin ortholog was present in another species, a reciprocal BLASTP search was performed. To perform this search, a gene of interest from the first organism is used in a BLAST search against a database of genes from the second organism. The top hit is then BLASTed against the first organism; if the top hit matches the original gene of interest, the sequences are considered to be orthologs. Dehydrins were only considered to be orthologs if the *E*-value was also <10^−10^. If an ortholog could not be found, the dehydrin DNA sequence was used in a BLASTN search against the species of interest genome for potential matches that may be located in regions considered to be non-coding sequences.

### Synteny analysis

The protein sequences of the first five genes that flank both sides of the dehydrin genes were collected and used to create a sequence database. BLASTP was used to search through the database of flanking genes using all sequences in the dehydrin database. Sequences that matched with an *E*-value of <10^−5^ were considered to be similar to each other.

## Results

**Fig 1:**
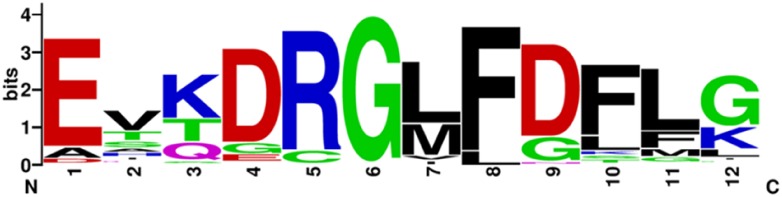
Logo representation of the F-segment discovered with MEME. The logo representation was generated using WebLogo [60]. The first three residues of the F-segment discovered in MEME were not very conserved, and were therefore removed for this representation. Colors represent each amino acid group: Black for hydrophobic (Ala, Ile, Leu, Met, Phe, Pro, Trp, Val), green for polar (Cys, Gly, Ser, Thr, Tyr), purple for neutral (Asn, Gln), red for negatively charged (Asp, Glu), and blue for positively charged (Lys, Arg, His).

After performing the filtering and iterative searching as described in the Material and Methods, a total of 426 dehydrin sequences were collected from 56 spermatophyte genomes. Of those dehydrins, 69 were the K_n_ architecture, 54 were K_n_S, 140 were SK_n_, 22 were Y_n_K_n_, and 141 were Y_n_SK_n_. In the three gymnosperm species, 22 K_n_ and 21 SK_n_ architectures were identified. Aside from the three well-documented segments (Y-, S- and K-segments), only two other motifs were consistently detected (i.e. in at least ~100 sequences). The logo of one motif can be seen in Fig 1, and is similar to the F-segment that was previously described by Strimbeck [59], but the two segment definitions differ at the termini. The F-segment described in Fig 1 has an additional Glu at the N-terminal end, followed by two fairly variable residues, while the F-segment described by Strimbeck had an additional two Lys at the C-terminal end [59]. The F-segment was found in 121 sequences, of which 111 were SK_n_ dehydrins and 10 were K_n_ dehydrins. The other motif that was detected is a string of Lys (11 residues of Lys interspersed with Arg, Asp and Glu), which was found in 94 sequences. In the 51 K_n_S dehydrins and 42 SK_n_ dehydrins, the poly-Lys repeat was located between the S- and K-segments. It was also detected in one K_n_ dehydrin.

**Fig 2:**
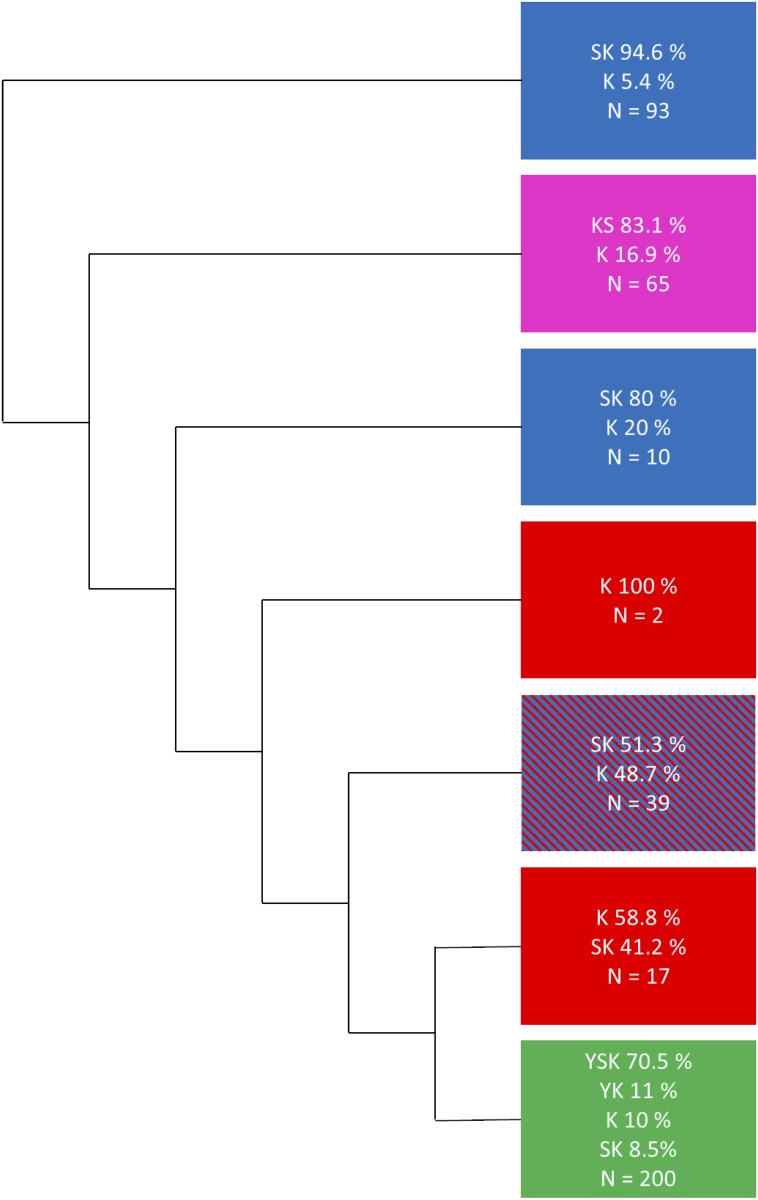
Simplified phylogenetic tree showing the major dehydrin architecture clusters. The phylogenetic tree in S1 Fig was reduced into clusters by visual inspection in order to minimize the number of clusters while trying to generate clusters that were enriched with just one architecture type. The colors represent the major architecture present in each cluster; red for K_n_ dehydrins, magenta for K_n_S dehydrins, blue for SK_n_ dehydrins, and green for Y_n_SK_n_ dehydrins.

The initial phylogenetic tree (Fig 2 and S1 Fig) had all of the K_n_S and Y_n_SK_n_ dehydrins fall into their own separate subtrees. The majority of the SK_n_ dehydrins did fall into one subtree, however, somewhat surprisingly, SK_n_ dehydrins were spread throughout the phylogenetic tree. All of the gymnosperm SK_n_ dehydrins fell outside of the subtree that contained the majority of the SK_n_ dehydrins, and were located between the K_n_S and Y_n_SK_n_ dehydrin subtrees. Some SK_n_ dehydrins were also present in the Y_n_SK_n_ dehydrin subtree. The Y_n_SK_n_ dehydrin subtree also contained all of the Y_n_K_n_ dehydrins, which were spread throughout the subtree. Lastly, the K_n_ dehydrins were present throughout the whole tree. Note that the bootstrap values identified for the large architecture splits were fairly low, with the highest being 20 for the K_n_S dehydrins.

Our goal was to determine which architectures likely evolved from another, but the scattering of some of the dehydrins among other architectures, and the low bootstrap values, were issues that needed to be considered first. With respect to the bootstrap values, the problem with dehydrins is that the conserved Y-, S-, K-segments are interspersed with poorly conserved ϕ-segments, making an accurate MSA a challenge. When a phylogeny is guided by a multiple sequence alignment, the quality of the alignment has a substantial impact on the resulting organization. A recently developed algorithm, called bubble clustering [61], has demonstrated a very high level of stability in constructing phylogeny and so rebuilding the phylogeny in the future with bubble clustering based on non-positional data may yield higher-quality results.

The other possibility, and the one we deal with here, is that some of the protein sequences are the result of sequence annotation errors. The sequences were analyzed to determine the typical number of exons present and the location of the conserved motifs with respect to the exon boundaries. Only angiosperm sequences were used for the analysis, since the gymnosperms data set consisted of only three species. Table 1 shows that most Y_n_SK_n_, Y_n_K_n_, and SK_n_ dehydrins have two exons, while K_n_S dehydrins mostly consist of one or two exons. In both Y_n_SK_n_ and SK_n_ dehydrins with two exons, the first exon ends with the S-segment, a feature previously identified by Jiménez-Bremont *et al*. [62].

**Table 1:** Comparison of dehydrin exon numbers in different architectures.

	Dehydrin Architecture				
Number of Exons	Y_n_SK_n_	Y_n_K_n_	SK_n_	K_n_S	K_n_
5+	3*	0	1	0	1
4	3	0	2	1	2
3	8	1	7	3	5
2	124	14	109	30	24
1	3	7	0	20	15

*See the text for an explanation.

Dehydrins with exons numbers higher than two and dehydrins with a different architecture than their neighbors in the initially generated tree (S1 Fig) were further inspected for possible sequence annotation errors. The non-coding regions flanking the dehydrin genes were investigated using JBrowser on Phytozome [45,63]. In cases where conserved motifs were detected in the putative intron of the dehydrin, or upstream or downstream of the dehydrin gene, the dehydrin sequence was reannotated to include the missing motifs. For cases where the segments were discovered in a non-coding region, but were separated from a dehydrin coding sequence by a stop codon, or if the segment was found to be out of frame, the sequence was recorded as a possible misannotation and the segment’s presence was documented. However, the protein sequence was left unchanged. The same was done for sequences where ambiguous nucleotides were present in the intron or present between a segment further upstream or downstream from the sequence of interest. Some dehydrin gene sequences were located near the ends of genome scaffold; this may have resulted in dehydrin sequences being split across two pieces of scaffold with only one part of the sequence being detected. Some sequences with more than the expected number of exons were reannotated to have two exons, but reannotation did not result in architectural changes. However, in two cases (marked with an asterisk in Table 1), the reannotation resulted in the dehydrin gene no longer being fused with a DNAJ or DNAJ-X domain containing gene.

As a result of the annotation error search, 30 sequences were reannotated and 28 sequences were detected as possible misannotations. Of the 30 reannotated sequences, 4 K_n_ dehydrins became SK_n_ dehydrins, 3 SK_n_ dehydrins became Y_n_SK_n_ dehydrins, and 1 K_n_ dehydrin became a K_n_S dehydrin, with the remaining 22 sequences not resulting in an architectural change. Within the 28 sequences that were possible misannotations (i.e. are not likely to be coding sequences), motifs were found that would make 4 K_n_ dehydrins become SK_n_ dehydrins, 2 SK_n_ dehydrins become Y_n_SK_n_ dehydrins, 1 K_n_ dehydrin become a KnS dehydrin, and 1 Y_n_K_n_ dehydrin become a Y_n_SK_n_ dehydrin. These changes brought the new total of each architecture to 59 K_n_, 56 K_n_S, 143 SK_n_, 21 Y_n_K_n_, and 147 Y_n_SK_n_. The F-segment was now found in 115 SK_n_ dehydrins and 6 K_n_ dehydrins. Of these 6 K_n_ dehydrins, 5 were in gymnosperms, the remaining one was in *Salix purpurea*.

**Fig 3:**
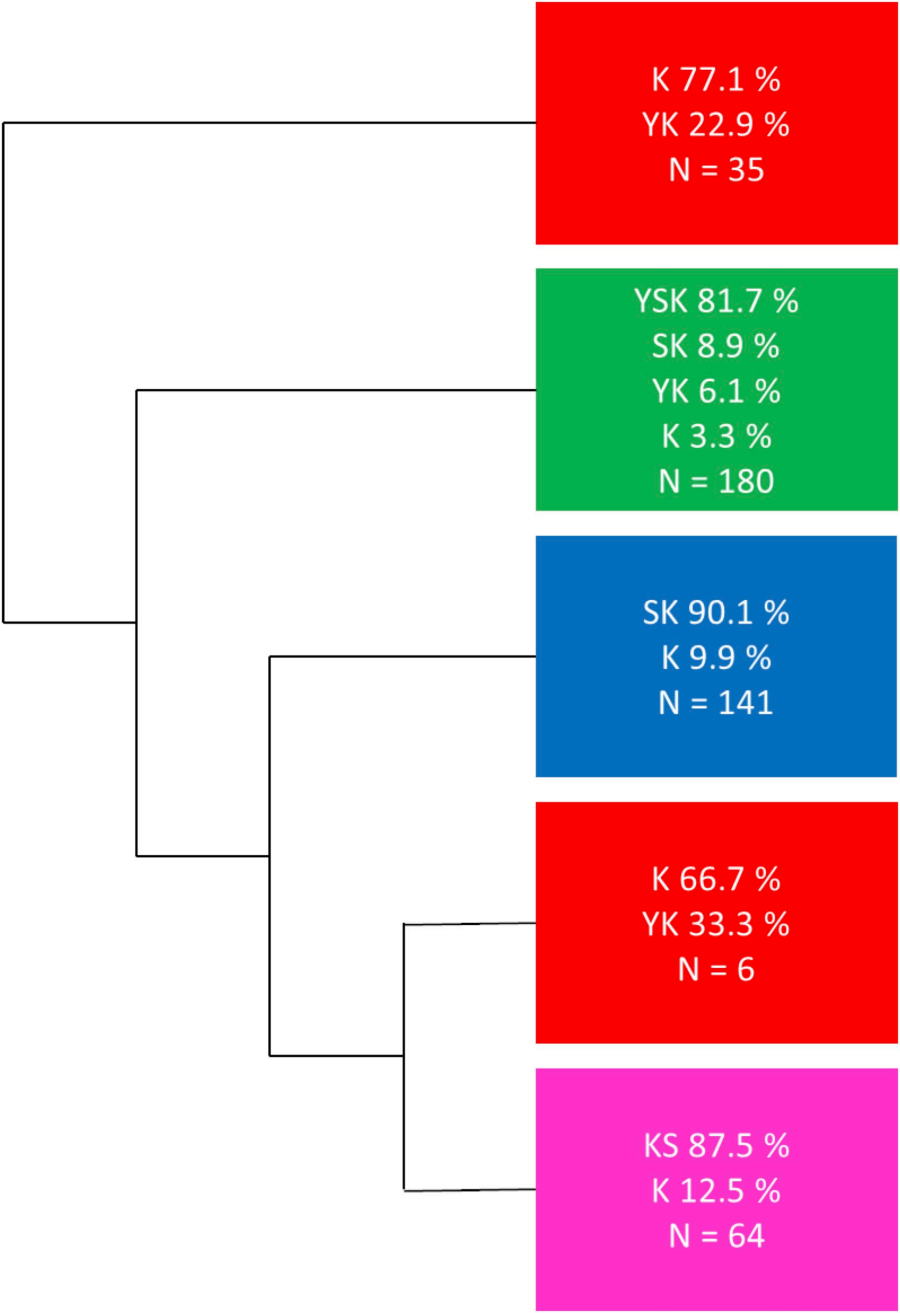
Simplified phylogenetic tree using reannotated sequences showing major dehydrin architecture clusters. The phylogenetic tree in S2 Fig was reduced into clusters by visual inspection in order to minimize the number of clusters while simultaneously trying to generate clusters that were enriched with just one architecture type. The colors represent the major architecture present in each cluster; red for K_n_ dehydrins, magenta for K_n_S dehydrins, blue for SK_n_ dehydrins, and green for Y_n_SK_n_ dehydrins.

A phylogenetic tree was then reconstructed including the 30 reannotated sequences. The newly constructed phylogenetic tree (Fig 3 and S2 Fig) has a more consistent separation of the different architectures, and could be divided into three major subtrees. The first major subtree contained all of the Y_n_SK_n_ dehydrins, as well as 6 K_n_, 16 SK_n_, and 11 Y_n_K_n_ dehydrins. None of the SK_n_ or K_n_ dehydrins in this subtree contained an F-segment. The second major subtree contained all of the K_n_S dehydrins, as well as 8 K_n_ dehydrins. The last major subtree contained all SK_n_ dehydrins with the exception of those in the Y_n_SK_n_ subtree, as well as 14 K_n_ dehydrins. Additionally, two smaller subtrees were present, both of which contained mainly K_n_ dehydrins with several Y_n_K_n_ dehydrins. However, the larger of these subtrees had a bootstrap value of 1, which suggests that this division is more than likely meaningless. The overall bootstrap values were again fairly low for the large architecture splits, however compared to the simplified tree in Fig 2 there is an improvement in the bootstrap values for the K_n_S and Y_n_SK_n_ dehydrin containing subtrees.

We subsequently examined whether the different architecture clusters had different levels of disorder using the FoldIndex tool [64]. The results (S3 Fig) are similar to those we previously observed for dehydrins when we clustered based on architecture [21]. Most of the architecture clusters have a similar amount of disorder, with the K_n_S dehydrin cluster appearing to be somewhat more disordered. It is not yet clear whether these differences have functional significance, or are more a reflection of the similar sequence composition of each of the different architectures.

**Fig 4:**
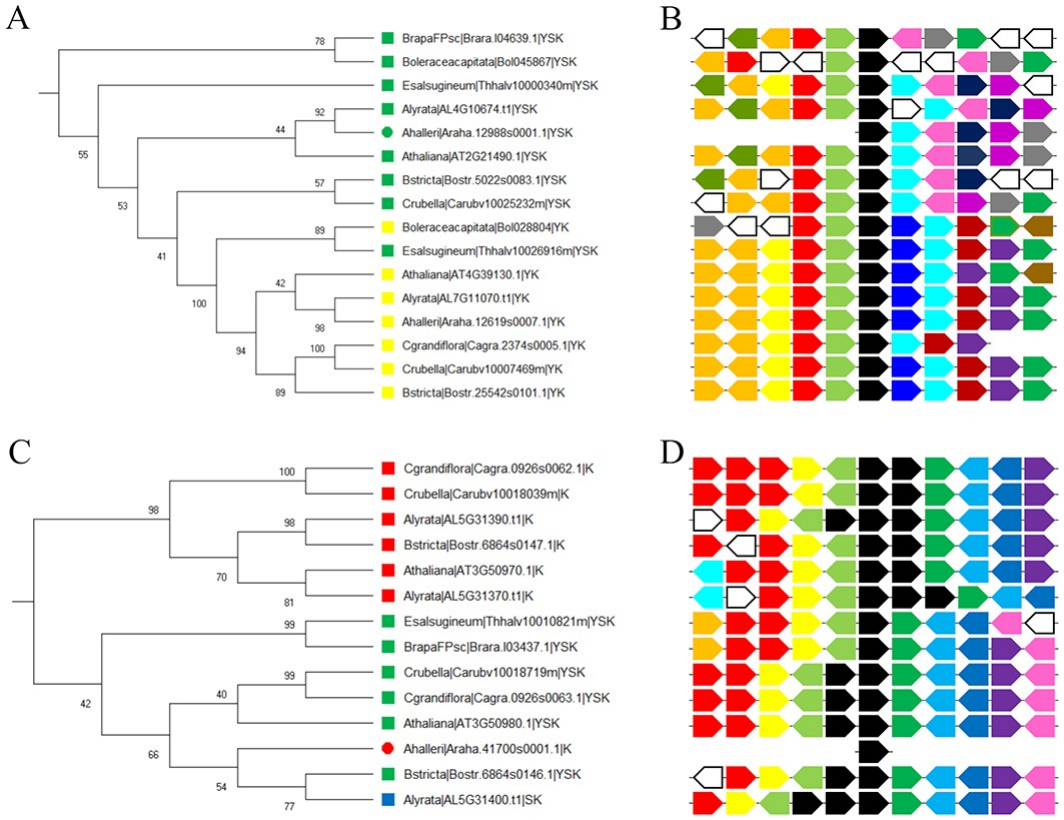
Subtrees with dehydrin architecture splits after gene duplication. **A)** Subtree of Y_3_K_1_ and Y_3_SK_2_ dehydrins with **B)** surrounding gene conservation diagram. This subtree was cut from the tree in S2 Fig. **C)** Subtree of K_6_ and Y_1_SK_2_ dehydrins with **D)** surrounding gene conservation diagram. This subtree was cut from the tree in S1 Fig. In the gene conservation diagram, black polygons represent the dehydrins, while polygons of the same color represent genes that were matched to each other by a BLAST search (*E*-value < 10^−5^). White polygons represent genes that did not have any matches. The direction the polygon points represents on which strand the gene is located. Polygons pointing right are on the forward strand while polygons pointing left are on the reverse strand.

The subtrees with bootstrap values >75 from both phylogenetic trees (S1 and S2 Figs) were inspected for large architecture divisions in order to detect possible evolutionary relationships between the dehydrins. Two subtrees of particular interest were further examined. The first subtree (Fig 4) contained all the Y_n_K_n_ dehydrins, as well as 9 Y_n_SK_n_ dehydrins, all from the family *Brassicaceae*. The Y_n_K_n_ dehydrins had three Y-segments and one K-segment; these dehydrins will from now on be referred to as the Y_3_K_1_ dehydrins. In comparison, the Y_n_SK_n_ dehydrins had three Y-segments and two K-segments, and will be referred to as the Y_3_SK_2_ dehydrins. One of the Y_n_SK_n_ dehydrins from *Eutrema salsugineum*, with the transcript ID Thhalv10026916m, had only one K-segment and a rather weak match to the S-segment (LRWFGISSANST). In the tree it was in the same clade as the Y_3_K_1_ dehydrins, so it was considered to be a Y_3_K_1_ dehydrin, with the assumption that the S-segment is in the process of being lost. The Y_3_K_1_ and Y_3_SK_2_ dehydrins were both present in *Arabidopsis thaliana*, *Arabidopsis lyrata*, *Capsella rubella*, *Boechera stricta*, *Brassica oleracea capitata*, and *Eutrema salsugineum*. Only the Y_3_K_1_ dehydrin was detected in *Capsella grandiflora*, however. The coding sequence for the Y_3_SK_2_ from *C*. *rubella* was used in a BLAST search against the *C*. *grandiflora* genome, and a fragment of 88 bp was found to be an excellent match with an *E*-value of 1.2 x 10^−19^. This sequence fragment was followed by an ambiguous nucleotide sequence, and therefore the whole sequence could not be established. *Brassica rapa* only contained the Y_3_SK_2_ dehydrin, so the coding sequence for the Y_3_K_1_ from *B*. *oleracea capitata* was used in a BLAST search against the entire *B*. *rapa* genome. There were no new dehydrin matches found in the non-coding sequences.

The genomes of species most closely related to *Brassicaceae* in the Phytozome (i.e. *Caprica papaya*, *Theobroma cacao*, *Gossypium raimondii*, *Citrus clementina*, and *Citrus sinensis*) were searched for orthologs to the Y_3_SK_2_ and Y_3_K_1_ dehydrins. Every species searched had one Y_n_SK_n_ dehydrin that was orthologous to either a Y_3_SK_2_ or a Y_3_K_1_ dehydrin from the subtree, however none of the species had an ortholog to both dehydrins. The sequences of all of the Y_3_SK_2_ and Y_3_K_1_ dehydrins were searched using BLAST against the genome of all the species of interest; however, no evidence of either dehydrin was found in coding or non-coding regions.

The second subtree of interest (Fig 4) was only observed in the tree in Fig 2 and S1 Fig. The subtree contained all but one of the K_n_ dehydrins in *Brassicaceae*, as well as six Y_n_SK_n_ and one SK_n_ architecture dehydrin. The K_n_ dehydrins all consisted of six K-segments with the exception of *A*. *lyrate*, which contained two K_n_ dehydrins (with five and six K-segments). These dehydrins will be referred to as the K_6_ architecture dehydrins. The Y_n_SK_n_ dehydrins all contained only one Y-segment and two K-segments; the SK_n_ dehydrins had a weak match to the Y-segment (DQFGIP) and had two K-segments. All of these dehydrins will be referred to as Y_1_SK_2_. Lastly, there was a K_n_ dehydrin with just two K-segments, however this *A*. *halleri* dehydrin gene was located near the end of a sequence fragment, so that the first exon may be present on a different fragment that could not be detected.

An important feature of this subtree is that all of the K_6_ dehydrins were found adjacent to the Y_1_SK_2_ dehydrins in the genome. In *A*. *thaliana*, *A*. *lyrata*, *C*. *rubella*, *C*. *grandiflora*, and *B*. *stricta*, both the K_6_ and Y_1_SK_2_ were present within ~600–1100 bp of each other. An additional K_5_ dehydrin was present in *A*. *lyrata* at the 5’ end of the K_6_ dehydrin (within ~3800 bp). *B*. *rapa* and *E*. *salsugineum* contained Y_1_SK_2_ dehydrins, but no orthologs to the K_6_ dehydrins were found and no matches were detected in the non-coding region of their genomes.

Although the exact subtree was not present in the phylogenetic tree in S2 Fig, there was a subtree that contained the same K_6_ dehydrins, and a subtree that contained the same Y_1_SK_2_ dehydrins.

The genes surrounding each of these dehydrins (Fig 4) were compared in order to investigate how the dehydrins are related to each other and to support the validity of the subtrees. In Fig 4B, it can be seen that genes surrounding the Y_3_K_1_ dehydrins are highly conserved, as well as the genes surrounding the Y_3_SK_2_ dehydrins. This would suggest that these dehydrins may have arisen after a large duplication event. The comparison of the genes surrounding the dehydrins from the K_n_/Y_n_SK_n_ tree shows that they are highly conserved (Fig 4D). The fact that the K_6_ and Y_1_SK_2_ dehydrins are adjacent to each other implies that these dehydrins most likely arose through a tandem duplication event.

**Fig 5:**
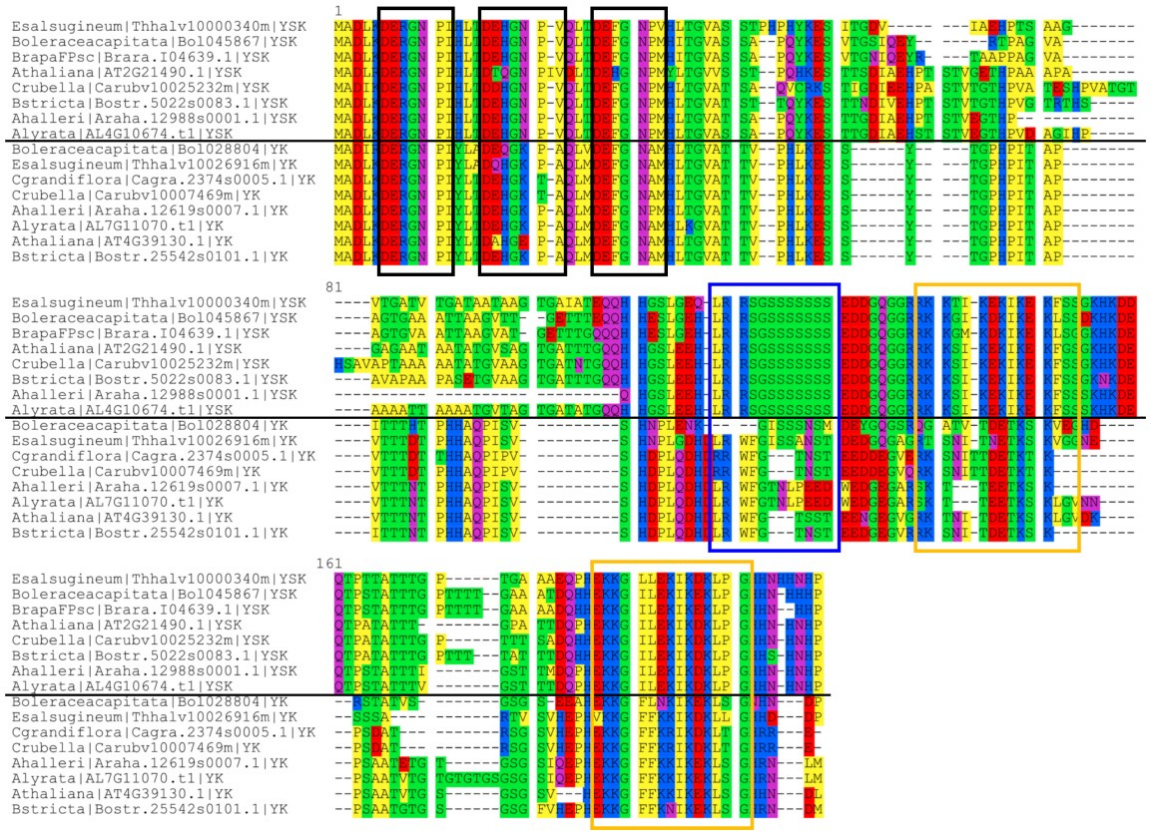
Multiple sequence alignment of Y_3_K_1_ and Y_3_SK_2_ dehydrins comparing their conserved segments. The multiple sequence alignment was generated by running MUSCLE with the default parameters, and was visualized using Seaview [52,54]. The conserved segments are shown bound by colored boxes. Black box, Y-segments; blue box, S-segments; orange box, K-segments. Colors represent the amino acid grouped by their chemical properties. Yellow, hydrophobic (Ala, Ile, Leu, Met, Phe, Pro, Trp, Val); green, polar (Cys, Gly, Ser, Thr, Tyr); purple, neutral (Asn, Gln); red, negatively charged (Asp, Glu); blue, positively charged (Lys, Arg, His).

A multiple sequence alignment of Y_3_SK_2_ and Y_3_K_1_ was created to compare the conserved motifs (Fig 5). The first of the three Y-segments is nearly completely conserved among all of these dehydrins, with the exception of the third position, which was usually Arg and occasionally Lys. The second Y-segment had the largest variability between the Y_3_SK_2_ and Y_3_K_1_ dehydrins, since the fifth position in the Y_3_SK_2_ dehydrins was always Asn and in the Y_3_K_1_ dehydrin was nearly always Lys. The third Y-segment is well conserved between the two architectures, with the exception of the sixth position, where Y_3_SK_2_ dehydrins contained a Pro and the Y_3_K_1_ dehydrins typically contained an Ala. The region of the Y_3_K_1_ dehydrins that aligns with the S-segment of the Y_3_SK_2_ dehydrins contained very few Ser residues, although it did end in several Asp/Glu residues, which is typical for this motif. However, the S-segment in Y_3_SK_2_ and the region of Y_3_K_1_ that aligns with the S-segment are both found at the end of the first exon of their respective genes, followed by the E/D sequence at the start of the second exon. The first K-segment from the Y_3_SK_2_ dehydrins aligns poorly with the Y_3_K_1_ dehydrins. In contrast, the second K-segment from the Y_3_SK_2_ dehydrin aligns well with the K-segment of the Y_3_K_1_ dehydrins, but does show a few differences. Positions 5–7 have the largest difference between the two; in the Y_3_SK_2_ dehydrins, the fifth position is usually occupied by an Ile, the sixth position is usually occupied by Leu, and seventh is usually occupied by Asp. In the same positions in the Y_3_K_1_ dehydrins, the K-segment has Phe in position five and six, and a Lys in position seven.

**Fig 6:**
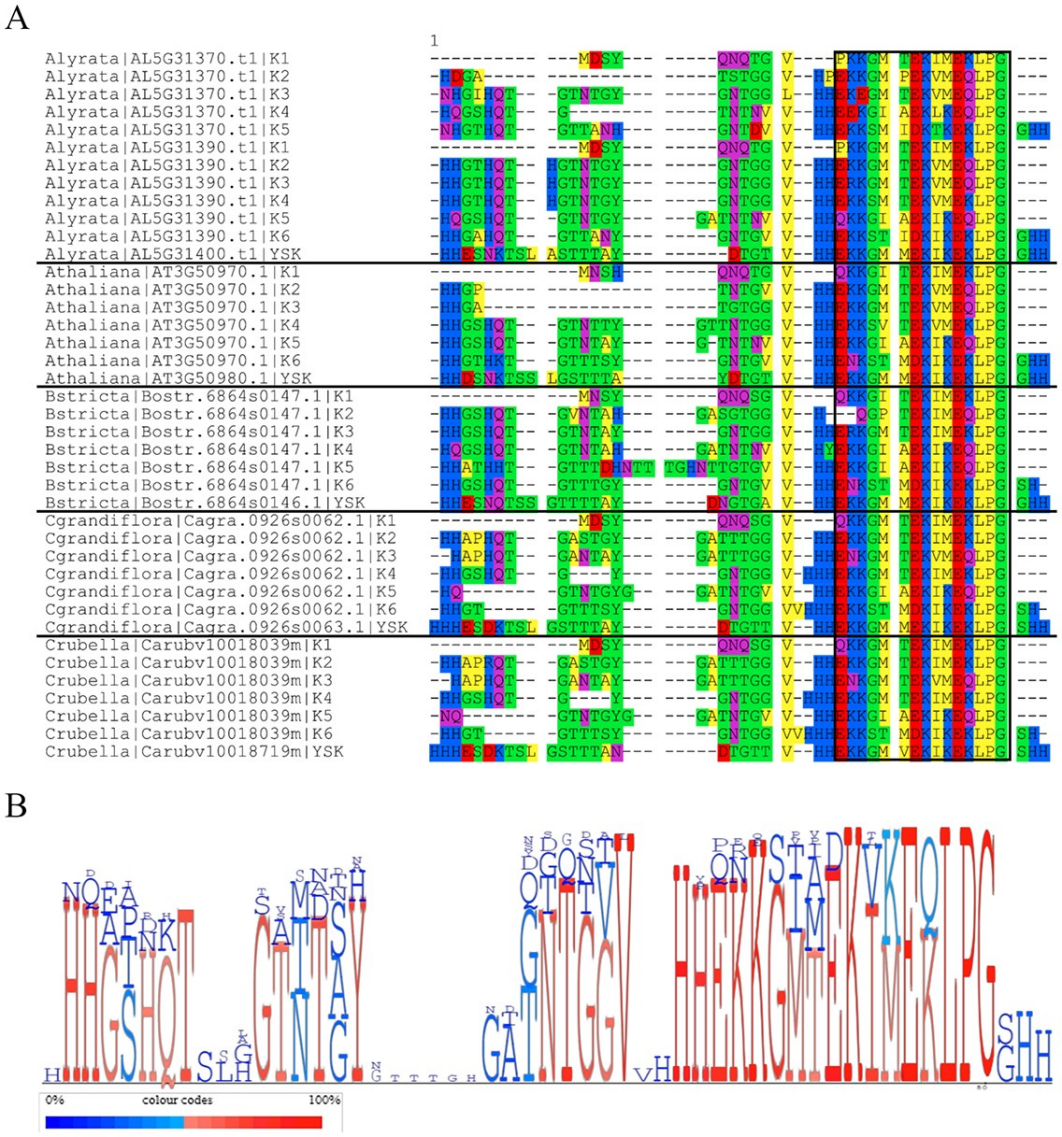
Multiple sequence alignment (MSA) of the K-segments with preceding ϕ-segments in the K_6_ dehydrin and the C-terminal K-segment with preceding ϕ-segment in the Y_1_SK_2_ dehydrin. **A)** MSA generated running MUSCLE with the default parameters, and visualized using Seaview [52,54]. The K-segment is shown bound by a black box. Amino acid colors are as described in Fig 5. **B)** Character height diagram generated using MulitDisp [65]. Red letters indicate residues that are conserved in more than 50% of the sequences while blue letters indicate residues that are conserved in less than 50% of the sequences.

We observed that the K-segments from the K_6_ dehydrins were highly similar in sequence. In Fig 6A, the K_6_ sequences were divided up such that the sequences were split after each K-segment, so that the K-segments and their preceding ϕ-segments could be aligned to each other. The last K-segment in the Y_1_SK_2_ dehydrins of their respective species was also included in the alignment along with the ϕ-segment up to the N-terminal K-segment. Fig 6B shows conservation of each position using character height. It shows that the K- and ϕ-segments are highly conserved, with the exception of gaps in the ϕ-segments.

**Fig 7:**
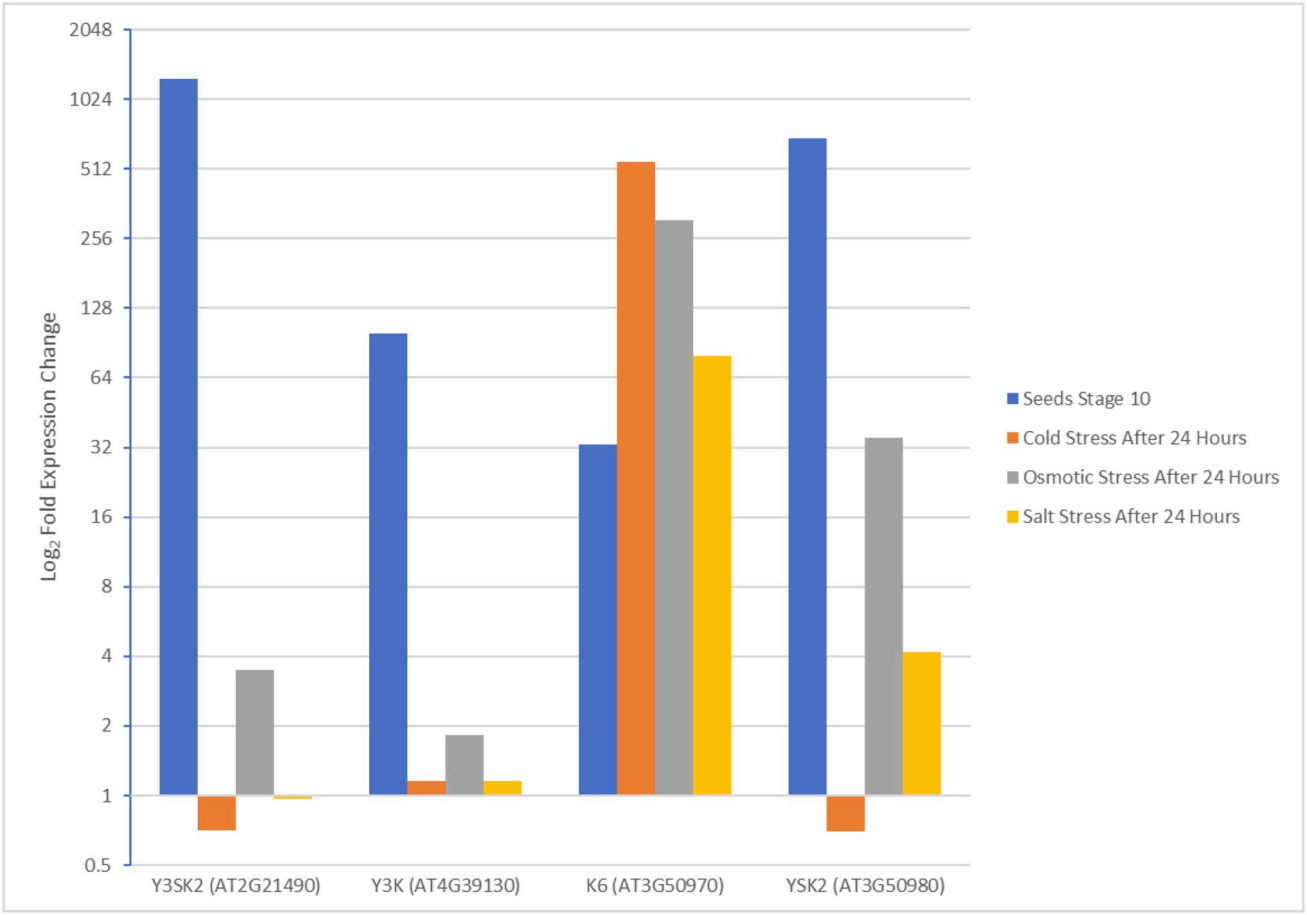
Bar graph comparing the fold-expression change of dehydrins from *Arabidopsis thaliana* shoots under abiotic stress and during seed development. Expression fold change values were collected from Arabidopsis eFP Browser at bar.utoronto.ca [66,67]. These values are the average of duplicate measurements.

Expression data for the dehydrins in *Arabidopsis thaliana* from the subtrees in Fig 4A and 4C were compared to help gain an understanding for what roles dehydrin duplicates might play in plant biology. Tissues with the highest expression fold changes from the development map and the abiotic stress map were selected from the Arabidopsis eFP Browser [66,67]. When comparing the expression fold change of the Y_3_SK_2_ and Y_3_K_1_ dehydrins (Fig 7 and S1 Table), it was found that both architectures share similar expression patterns. The highest expression fold change for both dehydrins was during late seed stages, however the expression level and the fold change were much higher for Y_3_SK_2_ (Fig 7 and S1 Table). Conversely, the Y_1_SK_2_ and K_6_ dehydrins showed very different expression patterns (Fig 7 and S1 Table). Under abiotic stress conditions, particularly under cold conditions, the K_6_ dehydrin experienced the most upregulation, while Y_1_SK_2_ dehydrin had the most upregulation during late seed stages.

## Discussion

Gene duplication is a major contributor to plant evolution. The average number of genes in plants found to be paralogous is 64.5%, ranging from 45.5% in *Physcomitrella patens* to 84.4% in *Malus domestica* [68]. There are five main mechanisms that result in gene duplication: whole genome duplication (WGD), tandem duplication, transposon-mediated duplication, segmental duplication, and retroduplication [68,69]. After duplication, a gene is either lost or retained; a gene can be lost if it undergoes pseudogenization or if it is deleted from the genome [68,69]. This can occur for various reasons, for example, if the duplicated gene is redundant and its loss results in no decrease in fitness [68,69]. A duplicated gene is retained if it results in improved fitness, such as from a change in function [68,69]. The presence of multiple dehydrins in different plant species [21] suggests that the different copies most likely arose through duplication events. The different dehydrin architectures that arose also suggest that dehydrins may be gaining new functions or variations of their original function. The only dehydrin architectures that were found across all spermatophytes we studied were the K_n_ and SK_n_ dehydrins. A lack of dehydrins containing the Y-segment in gymnosperms was previously described [70]. Expression data of Y-segment containing dehydrins was compared to SK_n_ in *A*. *thaliana*, *Populus trichocarpa*, *Oryza sativa*, *Zea mays*, and *Brachypodium distachyon* [66,71–74]. We observed that during seed germination, Y-segment containing dehydrins were always upregulated to very high levels, whereas SK_n_ dehydrins were not upregulated or only to a lower level (S2, S3, S4, S5 and S6 Tables). The absence of Y-segments in gymnosperms and the upregulation of Y-segment containing dehydrins in angiosperm seeds suggests the Y-segment has some role in coated seeds. Note that the F-segment was found in both angiosperm and gymnosperm SK_n_ dehydrins [59]. These findings give rise to the possibility that the architecture of the ancestral dehydrins in spermatophytes is either a K_n_ or SK_n_ dehydrin containing an F-segment, and that the Y_n_SK_n_ dehydrins arose after the angiosperms and gymnosperms division.

During the search for possible annotation errors in the dehydrin sequences, an apparent fusion of a dehydrin gene and a DNAJ and DNAJ-X containing gene was seen in *Brachypodium distachyon* and *Brachypodium stacei*. This fusion was also observed in *Oryza sativa* by Verma *et al*. [44]. DNAJ, also known as heat shock protein 40 (Hsp40), is a protein known to prevent the aggregation of unfolded protein and help fold proteins through its association with Hsp70 [75,76]. Heat shock proteins are synthesized during environmental stress, and overexpression of DNAJ in *Arabidopsis* has been shown to improve tolerance to NaCl [77]. The fact both dehydrins and DNAJ are associated with environmental stress and are in close proximity on the genome suggests the possibility there may be an association between the two. However, the sequence analysis performed here and the lack of experimental evidence to date suggests DNAJ and dehydrins are not expressed as a fusion.

The first phylogenetic tree (Fig 2 and S1 Fig) divided the K_n_S and Y_n_SK_n_ dehydrins into their own subtrees; unexpectedly the SK_n_ dehydrins were spread throughout the tree. The bootstrap values were also too low to reliably trust the data. The second phylogenetic tree (Fig 3 and S2 Fig) divided into major branches which contained primarily one architecture better than the first phylogenetic tree; these divisions for most part had improved bootstrap values as well. Unfortunately, it did not provide much information on where a split between the Y_n_SK_n_ dehydrins from either the K_n_ or SK_n_ dehydrins may have occurred, because, although improved, the bootstrap values were still too low to be considered reliable.

Although a high-level architecture source could not be reliably identified, two lower level architecture divisions were suggested by the data. The first is between the Y_3_K_1_ and Y_3_SK_2_ dehydrins. It was observed that nearly all the *Brassicaceae* species investigated contained both a Y_3_K_1_ dehydrin and Y_3_SK_2_ dehydrin that were present on different chromosomes (Fig 4A and 4B). A comparison of closely related species revealed that they all had only one ortholog to either Y_3_K_1_ dehydrin or Y_3_SK_2_ dehydrin, and none of them contained any other Y_n_K_n_ dehydrin. Based on these findings, it is likely that the Y_3_SK_2_ dehydrin was duplicated before the *Brassicaceae* lineage arose, but after the ancestor of *C*. *papaya* and the *Brassicaceae* family had split. Between these events, one whole genome duplication (WGD) event had occurred in the *Brassicaceae* lineage [68]. Known as the α WGD event, it is thought to have occurred approximately 47 million years ago [68,78]. Genes that arose from WGD events can be identified by regions of shared synteny around the duplicate genes. With the combination of the timing of the duplication and the observed conservation of the genes surrounding the Y_3_K_1_ and Y_3_SK_2_ dehydrins (Fig 4B), it is possible that the duplication of the Y_3_SK_2_ dehydrin occurred during the α WGD event.

After the duplication, one of the duplicated Y_3_SK_2_ dehydrins could have lost its S-segment and the N-terminal K-segment through mutations (Fig 5), which would result in these species gaining a dehydrin with the Y_3_K_1_ architecture. A comparison of the expression data of the Y_3_K_1_ dehydrin to the Y_3_SK_2_ dehydrin shows that they share a similar pattern of upregulation during seed development (Fig 7). However, the expression level observed in the Y_3_K_1_ dehydrins was much lower than in the Y_3_SK_2_ dehydrins (S1 Table), which can be an indicator this gene is on its way to becoming a pseudogene [79].

A second potential architecture split was identified between the K_6_ and Y_1_SK_2_ dehydrins within the *Brassicaceae* lineage (Fig 4C and 4D). The fact that these dehydrin genes are adjacent to one another in the genome is an indicator that they arose through a tandem duplication event [80]. Tandem duplications occur during unequal cross-over events [68]. Orthologs to either the K_6_ and Y_1_SK_2_ dehydrins were not detected in closely related species directly outside of *Brassicaceae*, and in *B*. *rapa* and *E*. *salsugineum*, only the Y_1_SK_2_ dehydrin was observed. This would suggest that the dehydrin present before duplication was Y_1_SK_2_, and that the tandem duplication occurred after *Arabidopsis* had separated from *B*. *rapa* and *E*. *salsugineum* (14 and 24 million years ago), but before the separation between *Arabidopsis* and *Capsella* lineages (between 10 and 14 million years ago) [81]. *A*. *lyrata* is the only species to contain an additional K_5_ dehydrin at the 5’ end of the K_6_ dehydrin. This K_5_ dehydrin may have arisen through an additional tandem duplication event.

In order for the duplicated Y_1_SK_2_ dehydrins to become K_6_ dehydrins, not only would the Y- and S-segment need to be lost, but also four K-segments would need to be gained. The observation that the K-segments in the K_6_ dehydrins were highly similar along with the ϕ-segments between them suggests they were repeated (Fig 6A). Out of 54 positions, 32 positions were conserved in 50% or more of all the sequences (Fig 6B). These K-segments and ϕ-segments were also observed to be very similar to the C-terminal region of the Y_1_SK_2_ region after the first K-segment, suggesting that it is the source of the duplication. It is possible that during DNA replication an error occurred that resulted in the C-terminal end in one of the Y_1_SK_2_ dehydrins being repeated multiple times, eventually becoming the K_6_ dehydrin. This was also suggested by Bies-Ethève *et al*. in their observations of the K_6_ and Y_1_SK_2_ dehydrins in *A*. *thaliana* [38]. When comparing the expression data of the K_6_ and the Y_1_SK_2_ dehydrins in *A*. *thaliana*, it was seen that the conditions under which the highest level of upregulation occur is different (Fig 7). The Y_1_SK_2_ was most upregulated during seed stage development, while the K_6_ dehydrin was most upregulated during abiotic stress, in particular cold stress. This would suggest that the duplication has led to neofunctionalization, where the Y_1_SK_2_ dehydrins are involved in the natural dehydration that occurs in seeds, while the K_6_ dehydrin is involved in cold stress protection in adult plants. In experiments done by Hughes *et al*. [25], it was found that the larger the number of K-segments, the better the cryoprotection. This suggests that the duplicated K_6_ gene may have been kept due to its advantage at protecting enzymes from cold damage.

## Conclusion

Two potential dehydrin architectural changes appeared after gene duplication. The first was a change from a Y_3_SK_2_ to a Y_3_K_1_ dehydrin, however the observed decrease in expression levels under similar conditions may be an indicator of the Y_3_K_1_ gene being pseudogenized. The second was a change from Y_1_SK_2_ to K_6_. The K_6_ dehydrin in *A*. *thaliana* had much higher expression during cold stress, and larger dehydrin constructs with higher numbers of K-segments have been shown to have improved enzyme cryoprotection [25]. These two duplications account for all of the K_n_ and Y_n_K_n_ dehydrins in the *Brassicaceae* family. Further work needs to be done to investigate what the architecture of the ancestral dehydrin was. Determining if and when the Y_n_SK_n_ dehydrins split from SK_n_ may be important in answering this question.

## Supporting information

S1 Fig Phylogenetic tree of 426 dehydrins from vascular plants. The tree was generated using RAxML with 100 bootstrap replicates. The architecture assignments are defined by the following coloring scheme: K_n_, red; K_n_S, magenta; SK_n_, blue; Y_n_K_n_, yellow; Y_n_SK_n_, green.
(PDF)

S2 Fig Phylogenetic tree of 426 dehydrins from vascular plants with reannotated sequences. The tree was generated using RAxML with 100 bootstrap replicates. The architecture assignments are defined by the following coloring scheme: K_n_, red; K_n_S, magenta; SK_n_, blue; Y_n_K_n_, yellow; Y_n_SK_n_, green. For the annotation analysis: ■, sequences that were unchanged; ▲, sequences that were reannotated; ●, sequences that were possible misannotations, unknown sequences, or pseudogenes. Unfilled symbols indicate dehydrin architectures that were changed by reannotation, or when motifs were found near dehydrins that were possible misannotations, unknown sequences, or pseudogenes.
(PDF)

S3 Fig Boxplot of disorder by architecture cluster. Dehydrin sequences were clustered as described for S2 Fig, and their level of disorder was predicted using the FoldIndex algorithm [64].
(PDF)

S1 Table Expression level and fold change of dehydrins from *Arabidopsis thaliana*. (PDF)

S2 Table Comparison of expression fold change of Y-segment containing dehydrins and SK_n_ dehydrins in *Arabidopsis thaliana* [60,61]. (PDF)

S3 Table Comparison of expression fold change of Y-segment containing dehydrins and SK_n_ dehydrins in *Populus trichocarpa* [65]. (PDF)

S4 Table Comparison of expression fold change of Y-segment containing dehydrins and SK_n_ dehydrins in *Zea mays* [60,66]. (PDF)

S5 Table Comparison of expression fold change of Y-segment containing dehydrins and SK_n_ dehydrins in *Oryza sativa* [67]. (PDF)

S6 Table Comparison of expression fold change of Y-segment containing dehydrins and SK_n_ dehydrins in *Brachypodium distachyon* [60,68]. (PDF)

S1 File All dehydrin coding sequences with reannotations. (FASTA)

S2 File All dehydrin peptide sequences with reannotations. (FASTA)

S3 File Dehydrin phylogenetic tree in Newick tree format. (NWK)

S4 File Dehydrin phylogenetic tree with reannotated sequences in Newick tree format. (NWK)

S5 File Reannotated dehydrin coding sequences. (FASTA)

S6 File Reannotated dehydrin peptide sequences. (FASTA)
