# Supplementary material for: Evolution of the modular, disordered stress proteins known as dehydrins
Source: PLoS One. 2019 Feb 6;14(2):e0211813. doi: 10.1371/journal.pone.0211813 (PMC6364937; doi:10.1371/journal.pone.0211813)
Supplement: S1 Fig — The tree was generated using RAxML with 100 bootstrap replicates. The architecture assignments are defined by the following coloring scheme: Kn, red; KnS, magenta; SKn, blue; YnKn, yellow; YnSKn, green. (PDF) [file pone.0211813.s001.pdf]

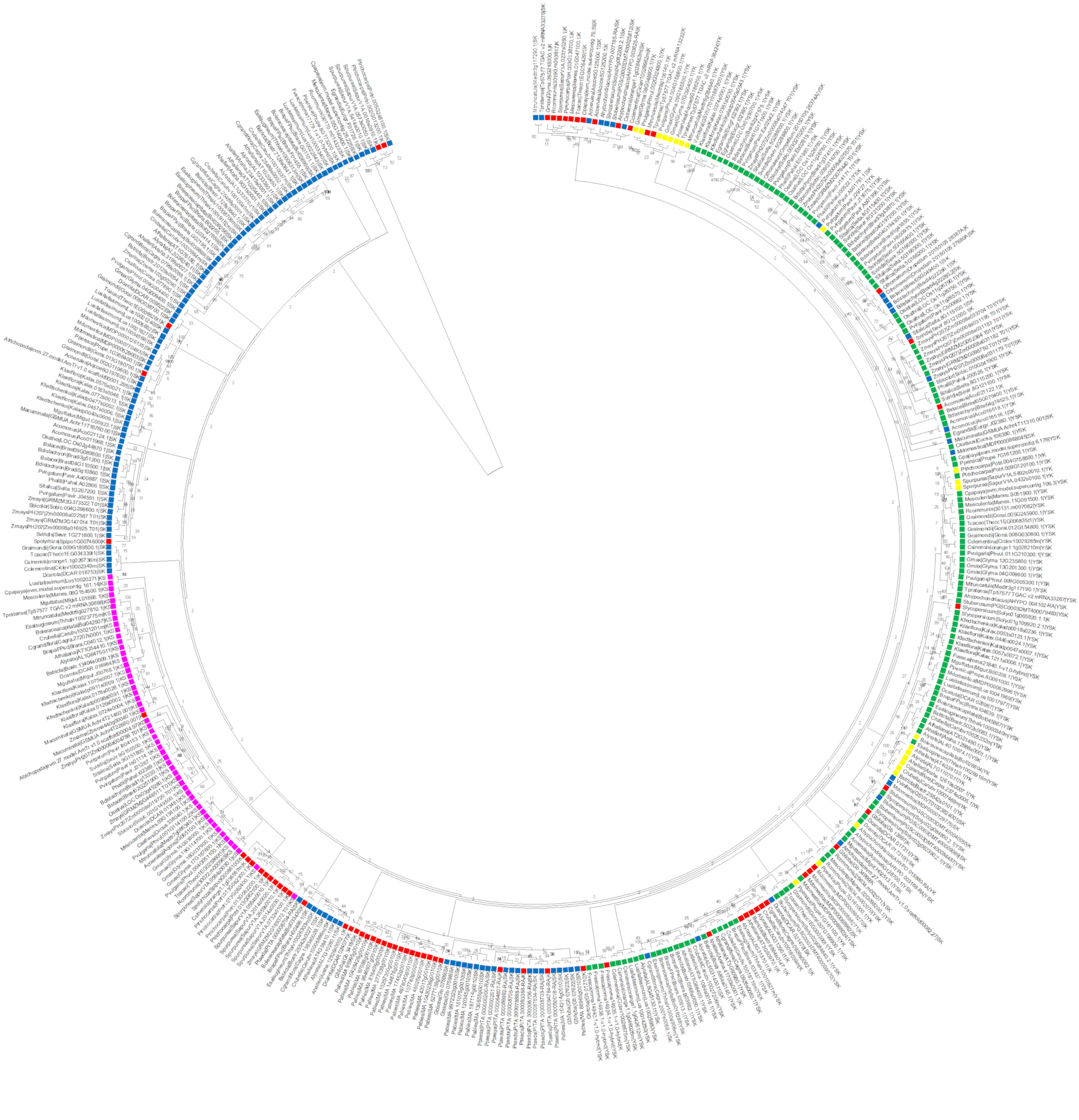

Figure S1. **Phylogenetic tree of 426 dehydrins from vascular plants.** The tree was generated using RAxML with 100 bootstrap replicates. The architecture assignments are defined by the following coloring scheme: Kn, red,; KnS, magenta; SKn, blue; YnKn, yellow; YnSKn, green.
