# Supplementary material for: Evolution of the modular, disordered stress proteins known as dehydrins
Source: PLoS One. 2019 Feb 6;14(2):e0211813. doi: 10.1371/journal.pone.0211813 (PMC6364937; doi:10.1371/journal.pone.0211813)
Supplement: S2 Fig — The tree was generated using RAxML with 100 bootstrap replicates. The architecture assignments are defined by the following coloring scheme: Kn, red; KnS, magenta; SKn, blue; YnKn, yellow; YnSKn, green. For the annotation analysis: ■, sequences that were unchanged; ▲, sequences that were reannotated; ●, sequences that were possible misannotations, unknown sequences, or pseudogenes. Unfilled symbols indicate dehydrin architectures that were changed by reannotation, or when motifs were found near dehydrins that were possible misannotations, unknown sequences, or pseudogenes. (PDF) [file pone.0211813.s002.pdf]

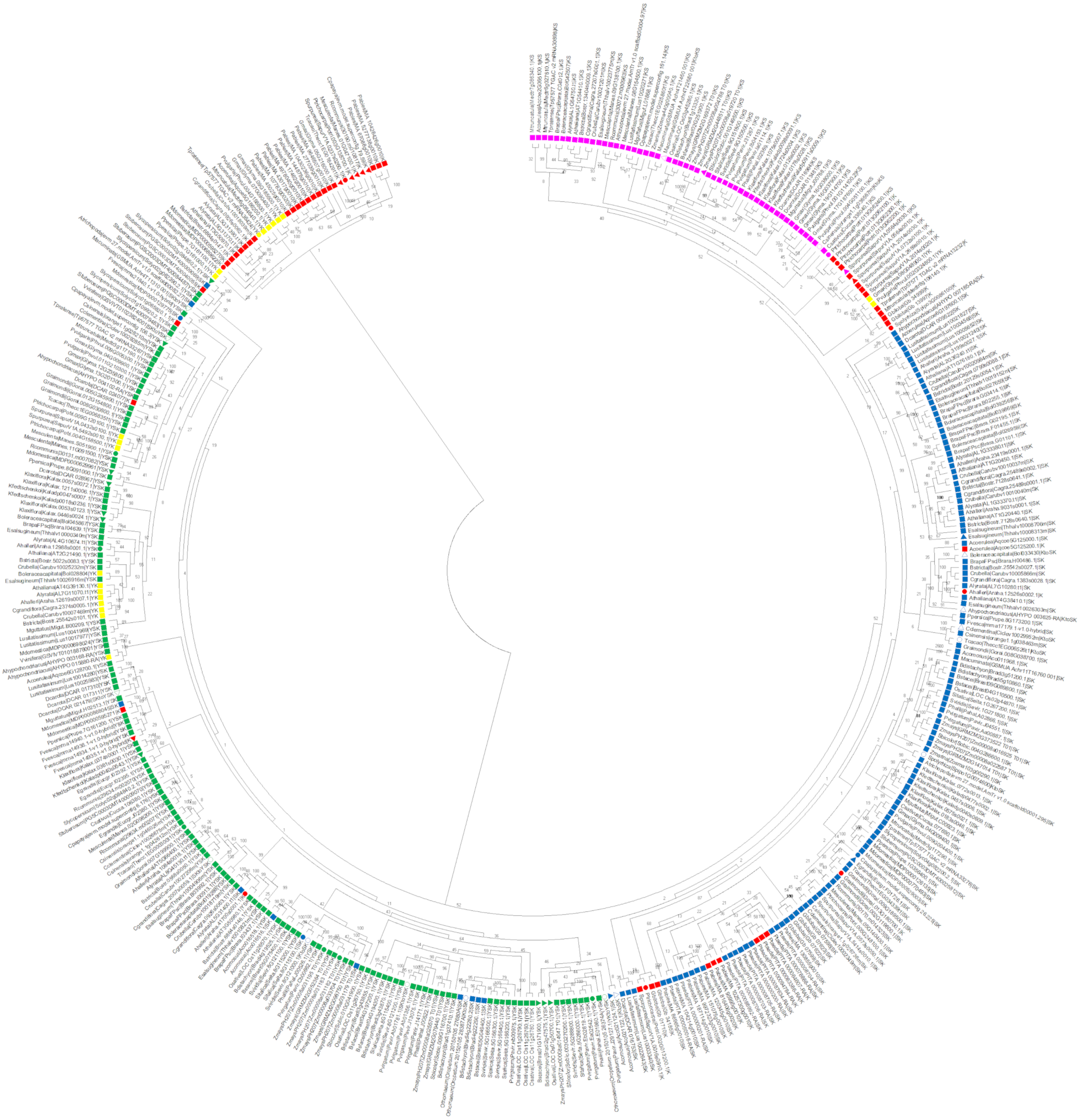

Figure S2. **Phylogenetic tree of 426 dehydrins from vascular plants with reannotated sequences.** The tree was generated using RAxML with 100 bootstrap replicates. The architecture assignments are defined by the following coloring scheme: Kn, red; KnS, magenta; SKn, blue; YnKn, yellow; YnSKn, green. For the annotation analysis: ■, sequences that were unchanged; ▲, sequences that were reannotated; ●, sequences that were possible misannotations, unknown sequences, or pseudogenes. Unfilled symbols indicate dehydrin architectures that were changed by reannotation, or when motifs were found near dehydrins that were possible misannotations, unknown sequences, or pseudogenes.
