# Supplementary material for: Evolution of the modular, disordered stress proteins known as dehydrins
Source: PLoS One. 2019 Feb 6;14(2):e0211813. doi: 10.1371/journal.pone.0211813 (PMC6364937; doi:10.1371/journal.pone.0211813)
Supplement: S3 Fig — Dehydrin sequences were clustered as described for S2 Fig, and their level of disorder was predicted using the FoldIndex algorithm [64]. (PDF) [file pone.0211813.s003.pdf]

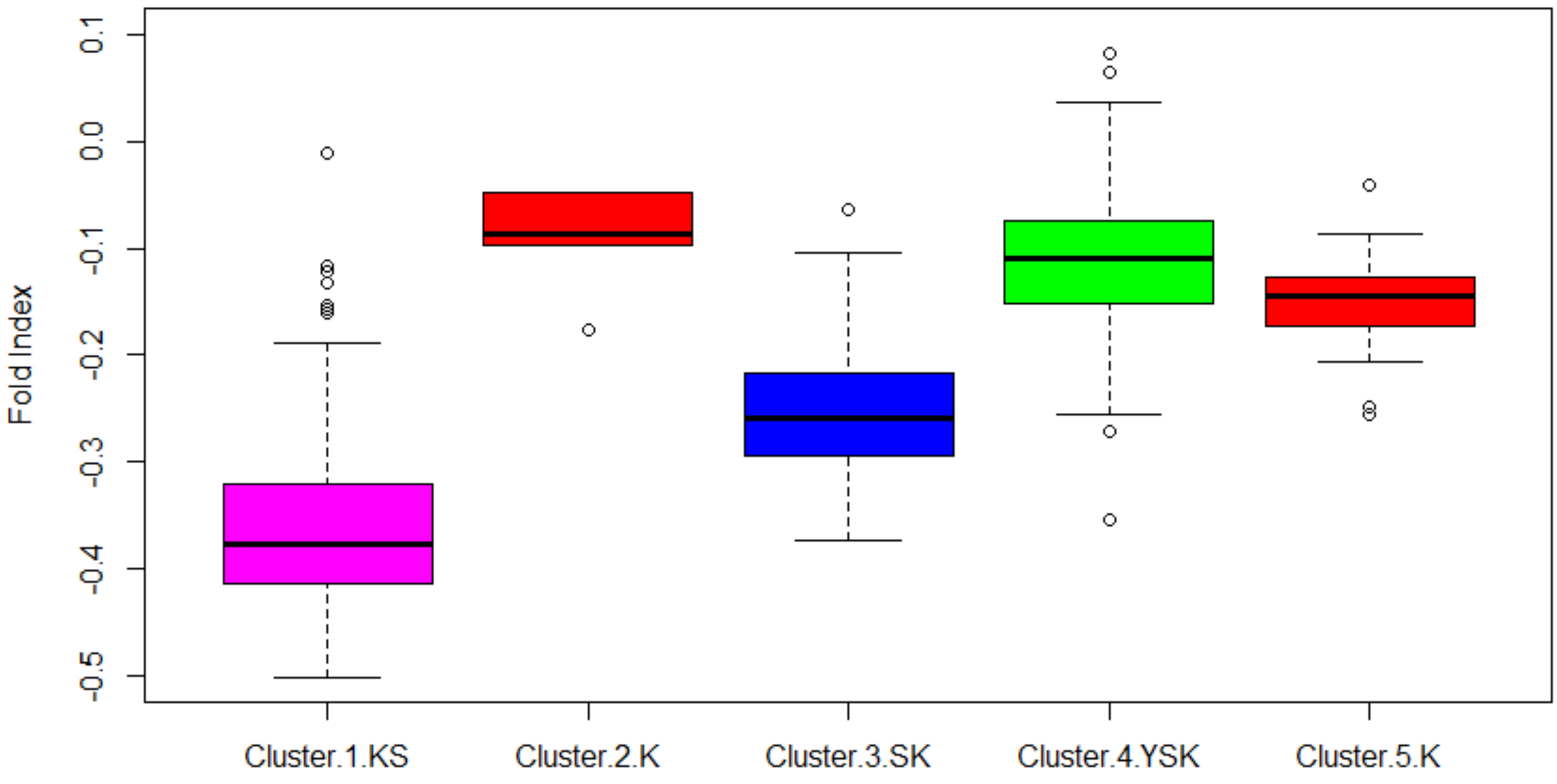

S3 Fig. Boxplot of disorder by architecture cluster. Dehydrin sequences were clustered as described for S2 Fig , and their level of disorder was predicted using the FoldIndex algorithm [64].
