# Supplementary material for: Evolution of the modular, disordered stress proteins known as dehydrins
Source: PLoS One. 2019 Feb 6;14(2):e0211813. doi: 10.1371/journal.pone.0211813 (PMC6364937; doi:10.1371/journal.pone.0211813)
Supplement: S1 Table — (PDF) [file pone.0211813.s004.pdf]

S1 Table. Expression level and fold change of dehydrins from *Arabidopsis thaliana*.

Expression Conditions	AT2G21490 Y_3SK_2		AT4G39130 Y_3K		AT3G50970 K_6		AT3G50980 YSK_2	
	Expression level	Fold Change	Expression level	Fold Change	Expression level	Fold Change	Expression level	Fold Change
Cold Shoot After 3 Hours	4.22	0.33	6.89	1.67	109.74	6.38	8.48	0.95
Cold Shoot After 6 Hours	6.15	0.56	5.43	0.77	1928.49	34.29	5.36	0.63
Cold Shoot After 12 Hours	12.08	0.97	7.29	1.26	5881.13	307.59	8.71	1
Cold Shoot After 24 Hours	6.3	0.71	4.85	1.15	8296.52	551.08	4.86	0.7
Osmotic Shoot After 3 Hours	4.99	0.39	6.6	1.6	901.77	52.48	11.3	1.27
Osmotic Shoot After 6 Hours	23.95	2.18	5.31	0.76	3449.73	61.35	26.25	3.08
Osmotic Shoot After 12 Hours	56.68	4.58	6.44	1.12	2882.35	150.75	87.14	10.03
Osmotic Shoot After 24 Hours	30.86	3.48	7.68	1.83	4643.46	308.43	242.78	35.05
Salt Shoot After 3 Hours	9.69	0.75	5.81	1.41	989.48	57.59	10.08	1.14
Salt Shoot After 6 Hours	4.83	0.44	8.4	1.2	1222.26	21.73	9.58	1.12
Salt Shoot After 12 Hours	9.59	0.77	5.85	1.01	527.48	27.58	9.26	1.06
Salt Shoot After 24 Hours	8.62	0.97	4.84	1.15	1198.19	79.58	29.04	4.19
Seeds Stage 6	1537.31	238.34	3.35	0.57	139.21	1.2	199.26	22.26
Seeds Stage 7	2488.43	385.8	14.48	2.49	970.35	8.37	520.76	58.18
Seeds Stage 8	6386.31	990.12	505.73	87.19	3224.63	27.83	5158.93	576.41
Seeds Stage 9	7812.06	1211.17	658.48	113.53	6382.25	55.09	6594.68	736.83
Seeds Stage 10	8131.25	1260.65	576.03	99.31	3830.73	33.06	6194.81	692.15
