# Supplementary material for: Evolution of the modular, disordered stress proteins known as dehydrins
Source: PLoS One. 2019 Feb 6;14(2):e0211813. doi: 10.1371/journal.pone.0211813 (PMC6364937; doi:10.1371/journal.pone.0211813)
Supplement: S2 Table — (PDF) [file pone.0211813.s005.pdf]

S2 Table. Comparison of expression fold change of Y-segment containing dehydrins and SK_n dehydrins in *Arabidopsis thaliana* [60,61].

	AT1G20440.1 SK_n	AT1G20450.1 SK_n	AT1G76180.1 SK_n	AT2G21490.1 Y_nSK_n	AT3G50980.1 Y_nSK_n	AT4G38410.1 SK_n	AT4G39130.1 Y_nK_n	AT5G66400.1 Y_nSK_n
Seeds Stage 6	0.37	1.08	0.65	238.34	22.26	8.86	0.57	3.5
Seeds Stage 7	0.98	2.05	1.04	385.8	58.18	9.79	2.49	26.38
Seeds Stage 8	0.11	0.27	0.58	990.12	576.41	2.74	87.19	241.12
Seeds Stage 9	0.22	0.41	0.92	1211.17	736.83	2.27	113.53	334.29
Seeds Stage 10	0.07	0.19	0.55	1260.65	692.15	1.8	99.31	330.87
