# Supplementary material for: Evolution of the modular, disordered stress proteins known as dehydrins
Source: PLoS One. 2019 Feb 6;14(2):e0211813. doi: 10.1371/journal.pone.0211813 (PMC6364937; doi:10.1371/journal.pone.0211813)
Supplement: S3 Table — (PDF) [file pone.0211813.s006.pdf]

S3 Table. Comparison of expression fold change of Y-segment containing dehydrins and SK_n dehydrins in *Populus trichocarpa* [65].

	Potri.004G158500.1 Y_nK_n	Potri.005G248100.1 SK_n	Potri.009G120100.1 Y_nSK_n
Dark-grown seedling, etiolated	7.36	1.65	30
Dark-grown seedling, etiolated, exposed to light for 3hr	36.21	1.3	112.59
Continuous light- grown seedling	5.92	0.81	22.33
