# Supplementary material for: Evolution of the modular, disordered stress proteins known as dehydrins
Source: PLoS One. 2019 Feb 6;14(2):e0211813. doi: 10.1371/journal.pone.0211813 (PMC6364937; doi:10.1371/journal.pone.0211813)
Supplement: S4 Table — (PDF) [file pone.0211813.s007.pdf]

S4 Table. Comparison of expression fold change of Y-segment containing dehydrins and SK_n dehydrins in *Zea mays* [60,66].

	GRMZM2G079440 Y_nSK_n	GRMZM2G147014 SK_n	GRMZM2G373522 SK_n
Germinating Seed 24h	130.19	0.2	0.69
