# Supplementary material for: Evolution of the modular, disordered stress proteins known as dehydrins
Source: PLoS One. 2019 Feb 6;14(2):e0211813. doi: 10.1371/journal.pone.0211813 (PMC6364937; doi:10.1371/journal.pone.0211813)
Supplement: S5 Table — (PDF) [file pone.0211813.s008.pdf]

S5 Table. Comparison of expression fold change of Y-segment containing dehydrins and SK_n dehydrins in *Oryza sativa* [67].

	LOC_Os01g50700.1 Y_nSK_n	LOC_Os02g44870.1 SK_n	LOC_Os11g26570.1 Y_nSK_n	LOC_Os11g26750.1 Y_nSK_n	LOC_Os11g26760.1 Y_nSK_n	LOC_Os11g26780.1 Y_nSK_n	LOC_Os11g26790.1 Y_nSK_n
Seed S1	2.09	2.22	2.12	13.05	1.77	1.28	2.41
Seed S2	9.82	1.67	2.44	9.87	2.91	8.86	5.46
Seed S3	161.43	1.37	87.69	30.18	38.25	19.04	18.69
Seed S4	404.79	0.71	332.45	119.49	151.8	88.57	40.19
Seed S5	426.81	0.14	222.35	231	203.58	117.87	41.57
