# Supplementary material for: Evolution of the modular, disordered stress proteins known as dehydrins
Source: PLoS One. 2019 Feb 6;14(2):e0211813. doi: 10.1371/journal.pone.0211813 (PMC6364937; doi:10.1371/journal.pone.0211813)
Supplement: S6 Table — (PDF) [file pone.0211813.s009.pdf]

S6 Table. Comparison of expression fold change of Y-segment containing dehydrins and SK_n dehydrins in *Brachypodium distachyon* [60,68].

	Bradi1g37410.1 Y_nSK_n	Bradi3g51200.1 SK_n	Bradi4g22280.2 SK_n	Bradi4g22290.1 SK_n	Bradi5g10860.1 SK_n
Endosperm 11 days after fertilization	1.14	0.16	10.21	3	2.3
Endosperm 31 days after fertilization	61.15	0.11	2.02	1.79	0.04
